# Nanoreactor Engineering Can Unlock New Possibilities for CO_2_ Tandem Catalytic Conversion to C—C Coupled Products

**DOI:** 10.1002/gch2.202300004

**Published:** 2023-05-01

**Authors:** Ali Goksu, Haitao Li, Jian Liu, Melis S. Duyar

**Affiliations:** ^1^ School of Chemistry and Chemical Engineering University of Surrey Guildford GU2 7XH United Kingdom; ^2^ State Key Laboratory of Catalysis Dalian Institute of Chemical Physics Chinese Academy of Sciences 457 Zhongshan Road Dalian 116023 China

**Keywords:** CO_2_ conversion, nano reactors, tandem catalysts

## Abstract

Climate change is becoming increasingly more pronounced every day while the amount of greenhouse gases in the atmosphere continues to rise. CO_2_ reduction to valuable chemicals is an approach that has gathered substantial attention as a means to recycle these gases. Herein, some of the tandem catalysis approaches that can be used to achieve the transformation of CO_2_ to C—C coupled products are explored, focusing especially on tandem catalytic schemes where there is a big opportunity to improve performance by designing effective catalytic nanoreactors. Recent reviews have highlighted the technical challenges and opportunities for advancing tandem catalysis, especially highlighting the need for elucidating structure‐activity relationships and mechanisms of reaction through theoretical and in situ*/*operando characterization techniques. In this review, the focus is on nanoreactor synthesis strategies as a critical research direction, and discusses these in the context of two main tandem pathways (CO‐mediated pathway and Methanol‐mediated pathway) to C—C coupled products.

## Graphical abstract

The level of carbon dioxide in the atmosphere is ever‐increasing and the environment is affected by this level. By using catalysis, the CO_2_ level can be reduced and useful chemicals and fuels can also be produced. This review focuses specifically on the use of tandem catalysts for CO_2_ conversion. Also, preferred nanotechnologies and catalysts for CO_2_ conversion are discussed.

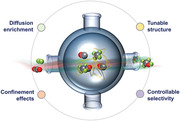

## 1 Introduction

**Figure 1:**
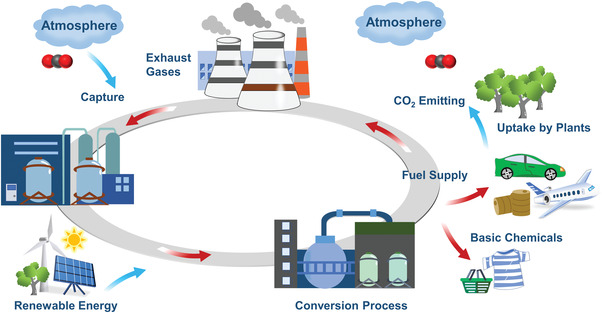
CO_2_ Recycling circular economy.

While the use of fossil fuels continues to dominate energy production, the atmospheric carbon dioxide (CO_2_) concentration has exceeded 400 ppm, posing a great threat to our environment through climate change and ocean acidification.^[^ ^1^, ^2^ ^]^ There is a constant increase in the concentration of CO_2_ in the atmosphere and this is associated with negative and irreversible effects on the world's climate. The chemical industry uses fossil resources as sources of carbon and hydrogen and to supply energy to drive reactions, releasing large amounts of greenhouse gases (CO_2_) in the process.^[^ ^3^ ^]^ As seen in **Figure** **1**, CO_2_ captured from industrial sources and atmospheric air can be used as an alternative carbon source and can be coupled to green hydrogen and renewable energy to implement sustainable chemical production and minimize environmental impacts.^[^ ^4^, ^5^, ^6^ ^]^ Such processes are needed urgently and at great scales to lower CO_2_ emissions associated with chemical production and establish a circular economy where carbon is recycled.^[^ ^7^ ^]^

CO_2_ capture and utilization (CCU) which involves the chemical conversion of captured CO_2_ is a potential pathway to a low‐emission industry.^[^ ^8^ ^]^ CCU is also appealing as a means to essentially recycle carbon in a circular economy. CO_2_ could be an alternative feedstock for producing carbon‐containing products and raw materials, many of which rely on fossil hydrocarbons for their production at present. CO_2_ is an abundant and geographically distributed carbon source that can be captured from industrial emission sources (such as cement manufacturing, and fossil‐based power stations) or adsorbed from the air via direct air capture to produce chemicals with high added value.^[^ ^2^, ^9^, ^10^ ^]^ CCU can both offer revenue to incentivize lowering CO_2_ emissions and provide a net‐zero emission solution to replace fossil carbon which is used at present for the synthesis of the world's chemical products.^[^ ^11^ ^]^

Many different CO_2_ conversion technologies are under development, including thermochemical, biological, electrochemical, and photoelectrochemical methods.^[^ ^12^ ^]^ Among these approaches, thermochemical catalytic hydrogenation of CO_2_ stands out as a mature technology with rapid kinetics and the potential for immediate deployment (if coupled with renewable energy‐driven production of green hydrogen).^[^ ^7^ ^]^ Electrochemical, photochemical, and biochemical approaches are also highly promising emerging pathways for converting CO_2_. Biological approaches such as algae growth have high operating costs but present the ability to produce long‐chain (C_2_–C_6_) hydrocarbons with high selectivity.^[^ ^13^ ^]^ Other microorganisms such as *Scenedesmus obliquus*, *Chlorella vulgaris*, *Synechococcus elongates*, and *Synechocystis sp*. produce valuable carbon products (ethanol, biodiesel, isoprene, ethylene, etc.) using CO_2_.^[^ ^14^ ^]^ Electrochemical approaches such as artificial photosynthesis and CO_2_ electrolysis offer the advantage of directly using renewable electricity to drive reactions.^[^ ^15^ ^]^ However, there are substantial process development requirements that need to be considered in CO_2_ utilization at scale, such as the reactor design for large‐scale photochemical systems, improving the lifetime of electrochemical catalysts, and controlling environmental conditions for biochemical systems.^[^ ^16^ ^]^ For example, changes on the catalyst surface in electrochemical reactions affect the stability of the system over time. These changes include contamination by metal impurities, surface poisoning by carbon species, and morphological changes of catalysts (dissolution, agglomeration, and coalescence of catalysts).^[^ ^17^ ^]^

In this review, we focus on thermal catalysts for CCU applications and highlight nanoreactor design as an approach that can achieve high selectivity, conversion rate, and stability. Nanoreactors are tiny reaction vessels that can be used to catalyze and control chemical reactions at the nanoscale level. The nanoreactor approach can enable the development of tandem catalytic systems where CO_2_ is converted into useful products such as fuels, chemicals, and other materials through multiple subsequent chemical transformations. In particular, nanoreactors can be envisioned as compartmentalized catalysts where CO_2_ is first reduced to CO or methanol, and then, the product of this reduction (CO/methanol) then diffuses into a different compartment of the nanoreactor with a different catalytic site for subsequent transformation (e.g., to C—C coupled products). Nanostructuring to create a “nanoreactor” allows to control the access of specific molecules to specific catalytic sites, thus also enabling control over the local reaction environment. This is a powerful synthetic tool to improve activity and selectivity in tandem catalytic systems, due to the greater degree of flexibility offered in terms of controlling local environment of distinct catalytic sites working in tandem.

Thermochemical catalytic hydrogenation processes can convert CO_2_ into single‐carbon (C_1_), or C—C coupled (C_2+_) products and they can be subject to lower barriers to increasing their technology readiness levels (TRL) compared to electrochemical and biological methods.^[^ ^12^, ^18^, ^19^ ^]^ Hydrogenation of CO_2_ with H_2_ from renewable energy sources not only offers a sustainable path to low or zero‐emission chemicals production but a means to store variable renewable energy in the form of chemical bonds, in an approach termed “power‐to‐X” (where X is the chemical produced).^[^ ^7^, ^20^ ^]^ There can be a lot of variations of this type of technology depending on what chemical (“X”) is targeted.^[^ ^21^, ^22^ ^]^ The conversion of CO_2_ to valuable products at an industrial scale requires selective catalysts with stable long‐term activity, and improved reactor and process technology.^[^ ^16^, ^23^ ^]^ While hydrogen is currently produced by steam reforming of methane which is a CO_2_ emitting method,^[^ ^24^ ^]^ projections indicate that green hydrogen produced by renewable energy‐driven water electrolysis will become cost‐competitive with fossil‐derived hydrogen in the coming decades.^[^ ^25^ ^]^

CO_2_ hydrogenation has been explored for the production of lower olefins, higher hydrocarbons, formic acid, methanol, and higher alcohols.^[^ ^26^, ^27^, ^28^, ^29^, ^30^, ^31^, ^32^ ^]^ Today, only 4% of total CO_2_ emissions are converted to chemicals (urea, methanol, salicylic acid, and organic carbonate).^[^ ^33^, ^34^ ^]^ The conversion of CO_2_ into liquid fuels such as methanol, gasoline, diesel, heating oil, or kerosene, can have a significant impact, as these products are responsible for 9Gt of CO_2_ equivalent emissions, or 30% of the total CO_2_ emitted today.^[^ ^35^ ^]^ Although there are many studies on the hydrogenation of CO_2_ to chemicals, few of these developed technologies have been designed and implemented commercially. The largest facility in operation today is the facility in Reykjavik, Iceland, owned by Carbon Recycling International (CRI), which produces 4000 t/year of methanol from 5500 t/year of CO_2_ conversion using heterogeneous catalysis and geothermal energy.^[^ ^36^ ^]^ The same group has also implemented a new commercial‐scale project in Anyang city, Henan Province, China. Here, methanol production is planned with a capacity of 110 000 tons/year from CO_2_ obtained from steel manufacturing.^[^ ^37^ ^]^ Finally, a methanol production facility was established in Niederaussem, Cologne, under the name of the pan‐European MefCO_2_ project (MefCO_2_, 2020) producing 365 tons of methanol and capturing more than 550 tons of CO_2_ annually.^[^ ^38^ ^]^

The most mature CCU technologies are the synthesis of urea, methane, and methanol. Urea synthesis is the main source of CO_2_ utilization and has been practiced for a long time. **Table** **1** shows the location and operational parameters of some of the plants from around the world that produce urea from CO_2_. Methanol synthesis using captured CO_2_ and renewable H_2_ is scaled up in the George Olah plant in Iceland and methanation of captured CO_2_ is being practiced in Germany at a large scale for the synthesis of e‐gas to power gas vehicles.^[^ ^39^, ^40^, ^41^, ^42^ ^]^

**Table 1:** Commercialized CCU plants for urea production, Reprinted with permission.^[^ ^39^ ^]^ Mitsubishi Heavy Industries.^[^ ^43^, ^44^ ^]^

Location	CO_2_ recovery capacity [metric ton/day]	Start of operation	Flue gas source
Kedah Darul Aman, Malaysia	160 (Max. 200)	October 1999	Natural gas‐fired steam reformer flue gas
Fukuoka, Japan	283 (Max. 330)	October 2005	Natural gas and heavy oil‐fired boiler flue gas
Aonla, India	450	December 2006	Natural gas‐fired steam reformer flue gas
Phulpur, India	450	December 2006	Natural gas‐fired steam reformer flue gas
Vijaipur, India	450	June 2012	Natural gas‐fired steam reformer flue gas
Kakinada, India	450	March 2009	Natural gas‐fired steam reformer flue gas
Bahrain	450	December 2009	Natural gas‐fired steam reformer flue gas
District Ghotoki, Pakistan	340	2011	Natural gas‐fired steam reformer flue gas
Phu My, Vietnam	240	2010	Natural gas‐fired steam reformer flue gas
Perm, Russia	1200	2021 (Under Construction)	Natural gas‐fired steam reformer flue gas
Polash, Narsingdi, Bangladesh	240	2023 (Under Construction)	Natural gas‐fired steam reformer flue gas

Methanol is also very important in the chemical industry and is considered a solvent, raw material, and energy source of the future.^[^ ^45^ ^]^ Mixed metal oxides (Cu—ZnO—Al_2_O_3_), which are used to synthesize methanol industrially from mixed synthesis gas (CO/CO_2_/H_2_), show low yields and poor activity for CO_2_/H_2_ feeds due to the generation of significant quantities of water in the reaction.^[^ ^46^ ^]^ CO_2_ methanation is also a mature process, and catalysis is carried out by using transition metals such as Co, Ni, Ru, Rh, and Pd. Co and Ni‐based catalysts are preferred more than noble metals (Ru, Rh, Pd) due to their low cost.^[^ ^47^, ^48^ ^]^ Ni‐based catalysts are the most widely used industrially because they have high activity, high CH_4_ selectivity, and earth abundance.^[^ ^49^, ^50^ ^]^ In addition to these technologies, the production of synthesis gas (syngas), a mixture of carbon monoxide (CO), and H_2_, from CO_2_ is possible via the reverse water gas shift reaction (RWGS). Syngas is of interest as a product of CCU because commercial pathways^[^ ^51^ ^]^ from syngas exist for the production of a host of hydrocarbon products through the Fischer–Tropsch process.^[^ ^52^, ^53^, ^54^ ^]^

Direct conversion of CO_2_ to C—C coupled products in a single reactor is appealing due to the opportunity that offers in decarbonizing the broader chemical industry. It is very difficult to find active, selective, and stable catalysts in the production of olefins by the CO_2_ hydrogenation reaction. This can be explained as follows: there are 3 ways to produce olefins from CO_2_. These include 1) direct production with promoted catalysts; 2) methanol production followed by olefin synthesis (MeOH‐mediated route); 3) CO production with RWGS followed by olefin synthesis with Fischer‐Tropsch synthesis (FTS).

**Figure 2:**
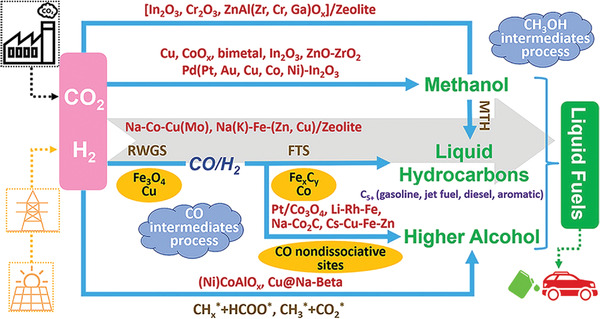
Summary schematic representation for the methods and catalysts used in the production of fuel from CO_2_, Reproduced with permission.^[^ ^36^ ^]^ Copyright 2020, ACS Publications.

FTO, MeOH synthesis, and CO_2_ methanation are exothermic while RWGS is an endothermic process. Accordingly, it is understood that lower temperatures favor FTO, MeOH synthesis, and methanation, but higher temperatures can achieve fast reaction rates. Tandem catalysis is recently being explored as a way of achieving higher selectivity at a lower cost through the efficient combination of reactions.^[^ ^1^ ^]^ The hydrogenation of CO_2_ to C_2+_ hydrocarbons is very important because long‐chain hydrocarbons have higher energy density and can be used as fuel or chemicals in different fields.^[^ ^55^, ^56^, ^57^ ^]^ In **Figure** **2**, some preferred catalysts and reactions in fuel production from CO_2_ are explained. There are two ways to hydrogenate CO_2_ to hydrocarbons; these are direct and indirect ways.^[^ ^58^ ^]^ One approach that has gathered much attention is the development of tandem catalysis schemes to diversify the processes for CO_2_ utilization; these schemes combine catalysts with different functions to achieve a multistep conversion of CO_2_ in the same reactor to products like olefins and alcohols. For the tandem approach to be selective, we need careful control of the microenvironment, selective access of certain molecules to specific active sites, and tunable placement and properties of active sites. Nanoreactors are materials that are structured to offer such controlled properties and catalytic behavior, which is the subject of this review, with a focus on their development for tandem catalytic conversion of CO_2_ to chemicals. Tandem CO_2_ conversion refers to the process of converting CO_2_ into useful products in multiple steps or stages, where the products of one reaction are used as the reactants for subsequent reactions.^[^ ^59^, ^60^ ^]^ Tandem reactions have the potential to achieve higher conversion rates and selectivities compared to single‐step reactions, as well as to generate more valuable products.^[^ ^61^ ^]^Tandem CO_2_ conversion processes have been demonstrated in thermochemical as well as electrochemical approaches. One example of a thermochemical tandem catalytic CCU process is where CO_2_ is first converted to CO via hydrogenation (RWGS), and then the CO further reacts on a separate catalytic site with hydrogen to produce hydrocarbons or other chemicals.^[^ ^62^, ^63^ ^]^ In electrochemical approaches CO_2_ reduction to CO can be achieved directly, without hydrogen feed.^[^ ^64^, ^65^, ^66^, ^67^ ^]^ The resulting CO can be electrochemically converted into useful chemicals such as alcohols or olefins on a different active site of the catalyst.^[^ ^68^, ^69^ ^]^

Overall, tandem CO_2_ conversion is a promising strategy for converting CO_2_ into useful products and mitigating the negative effects of greenhouse gas emissions on the environment.

Recently, many researchers/research groups are discussing various aspects of tandem CO_2_ conversion, including the design^[^ ^70^ ^]^ and optimization of catalysts,^[^ ^71^ ^]^ the identification of new reaction pathways and products, and the challenges associated with scaling up the processes.^[^ ^72^, ^73^ ^]^ They also highlight the potential of tandem CO_2_ conversion to contribute to the development of sustainable and low‐carbon technologies.^[^ ^74^, ^75^ ^]^

Nanoreactor catalysts can offer superior performance and extended life over conventional catalysts for thermal CO_2_ hydrogenation. Nanoreactor engineering refers to the design and construction of nano‐sized reactors that can be used for various chemical reactions, including CO_2_ conversion.^[^ ^76^ ^]^ CO_2_ conversion achieves the recycling of carbon dioxide into useful chemical products, such as fuels, chemicals, or materials.^[^ ^77^, ^78^ ^]^ Nanoreactors can be used to enhance the efficiency and selectivity of CO_2_ conversion reactions by providing a highly controlled environment for the reaction to take place.^[^ ^79^ ^]^

Nanoreactors typically consist of a nano‐sized container or cavity, which can be filled with a catalyst and/or reactant.^[^ ^80^ ^]^ The size and shape of the nanoreactor can be tailored to optimize the reaction conditions, such as temperature, pressure, and reactant concentration.^[^ ^81^, ^82^ ^]^ In addition, the nanoreactor can be designed to enhance the interaction between the catalyst and reactants, improving the reaction efficiency and selectivity.^[^ ^83^ ^]^

One approach to nanoreactor engineering for CO_2_ conversion is the use of metal‐organic frameworks (MOFs), which are porous materials made of metal ions and organic ligands.^[^ ^84^, ^85^ ^]^ MOFs can be designed with specific pore sizes and structures, allowing for the precise control of reactant diffusion and reaction kinetics.^[^ ^86^ ^]^ They can also be functionalized with specific catalytic sites to enhance the reaction efficiency and selectivity.^[^ ^87^ ^]^ Another approach is the use of nanoparticles as catalytic sites within the nanoreactor. Nanoparticles can provide a high surface area for reactant adsorption and catalytic activity, and can be engineered with specific shapes and compositions to optimize the reaction conditions.^[^ ^88^ ^]^

Overall, nanoreactor engineering offers a promising approach to CO_2_ conversion, as it allows for the precise control of reaction conditions and catalytic activity, leading to improved efficiency and selectivity.^[^ ^89^ ^]^ Recent review articles highlight the uses of nanoreactor engineering for CO_2_ conversion, focusing specifically on carbon‐based systems, porphyrins, and yolk‐shell structures, discussing recent advances and future prospects relevant to these syntheses.^[^ ^90^, ^91^, ^92^, ^93^, ^94^, ^95^ ^]^ Herein we focus on applications relevant to tandem catalysis.

There are many advantages of using nanoreactors in CO_2_ conversion, some of which can be explained as follows: 1) Increased catalytic efficiency: Nanoreactors can increase the catalytic efficiency of CO_2_ conversion by providing a high surface area for the catalyst to interact with CO_2_. This can lead to higher reaction rates and improved product selectivity.^[^ ^94^, ^96^ ^]^ 2) Precise control over reaction conditions: Nanoreactors can provide precise control over the reaction conditions, such as temperature, pressure, and pH, which can optimize the conversion process and improve product yield.^[^ ^97^ ^]^ 3) Reduction in energy requirements: Nanoreactors can reduce the energy requirements for CO_2_ conversion by providing a confined reaction environment that can facilitate the reaction at lower temperatures and pressures.^[^ ^98^ ^]^ 4) Selective product formation: Nanoreactors can be designed to selectively convert CO_2_ into specific products, such as methanol or other value‐added chemicals, which can increase the economic viability of the process.^[^ ^99^ ^]^ 5) Versatility: Nanoreactors can be used for a variety of CO_2_ conversion processes, including thermochemical, electrochemical, and photocatalytic reactions, allowing for a range of options for CO_2_ conversion.^[^ ^100^, ^101^ ^]^

This review is aimed at presenting the current status of the development of nanoreactor catalysts for hydrocarbon production by thermal CO_2_ hydrogenation. We begin by highlighting some promising CO_2_ hydrogenation schemes and proceed to motivate the development of nanoreactor architectures that can achieve selective tandem catalytic conversion by employing some of these pathways.

## 2 High‐Value Production Methods from CO_2_ Conversion

### 2.1 Two Step CO_2_ Hydrogenation with RWGS + FTS

With thermochemical hydrogenation methods, CO_2_ can be converted to one‐carbon (C1) products such as methane (Equation (1)), methanol (Equation (2)), and carbon monoxide (Equation (3)) in a single‐step process.^[^ ^102^ ^]^ It is possible to use the RWGS reaction (Equation (2)) as an initial step for conversion of CO_2_ to C—C coupled products, by performing the FTS reaction (Equation (4)) in tandem.^[^ ^58^ ^]^

(1) $$\begin{array}{c} {\mathrm{CO}}_{2}+4H_{2}↔{\mathrm{CH}}_{4}+2H_{2}O,\;\Delta H_{298}^{0}=-164\;{\mathrm{kj}\;\mathrm{mol}}^{-1} \end{array}$$

(2) $$\begin{array}{c} {\mathrm{CO}}_{2}+3H_{2}↔{\mathrm{CH}}_{3}\mathrm{OH}+H_{2}O,\;\Delta H_{298}^{0}=-49\;{\mathrm{kj}\;\mathrm{mol}}^{-1} \end{array}$$

(3) $$\begin{array}{c} {\mathrm{CO}}_{2}+H_{2}↔\mathrm{CO}+H_{2}O,\;\Delta H_{298}^{0}=+41\;{\mathrm{kj}\;\mathrm{mol}}^{-1} \end{array}$$

(4) $$\begin{array}{c} \mathrm{CO}+2H_{2}↔-\left({\mathrm{CH}}_{2}\right)-+H_{2}O,\;\Delta H_{298}^{0}=-166\;{\mathrm{kj}\;\mathrm{mol}}^{-1} \end{array}$$

It is possible to perform CO_2_ hydrogenation to CO followed by FTS in two reactors, where the first reactor would operate at high temperatures (200–600 °C) and intermediate pressure (0–20 bar) conditions^[^ ^103^, ^104^ ^]^ for RWGS and the second (FTS) reactor is operated at a temperature of 200 to 300 °C and a pressure of 10 to 40 bar.^[^ ^105^ ^]^ Because RWGS is an endothermic reaction, high temperatures are required for high conversions.^[^ ^106^, ^107^ ^]^ Tandem catalysis schemes seek to perform both RWGS and FTS reactions in a single reactor by coupling an RWGS catalyst with an FTS catalyst and operating at a range of conditions that compromise between the two reactions.^[^ ^104^ ^]^ A tandem catalyst required to produce C_2+_ products (light olefin, liquid fuel, or higher alcohol products) from CO_2_ must be active for both RWGS and FTS under the same conditions to be effective.^[^ ^55^, ^108^ ^]^ It is proposed that consumption of the product of RWGS (CO) by the FTS reaction can lead to additional synergies in operation.

### 2.2 Catalysts for the RWGS Reaction

Various catalytic materials have been used in research to carry out the RWGS reaction and several reviews have covered the development of these catalysts in recent years.^[^ ^109^, ^110^, ^111^, ^112^ ^]^ Regarding the noble metal‐based catalysts, the most widely used are the supported Pt catalysts,^[^ ^113^, ^114^ ^]^ which exhibit significant activity and selectivity for the hydrogenation of CO_2_ to CO.^[^ ^110^, ^115^, ^116^, ^117^, ^118^ ^]^ **Table** **2** contains details on CO_2_ conversion and CO selectivity of some RWGS catalysts under certain reaction conditions.

**Table 2:** Performance of selected catalysts for the RWGS reaction

Catalyst	Temperature [°C]	Pressure [MPa]	CO_2_ conversion [%]	CO selectivity [%]	WHSV [mLg_cat_^−1^ h^−1^]	Reference
2% Pt/CeO_2_	225	N/A	13.7	99	N/A	[119]
Ru‐Cu/ZnO/Al_2_O_3_	500		47	100	100 000	[120]
K/Mn/Fe/ Al_2_O_3_	300 °C	1.83	44.7	47.3	N/A	[121]
K‐Mo_2_C/*γ*‐Al_2_O_3_	450 °C	2.1	44	98	6120	[51]
Cu/*β*‐Mo_2_C	600 °C	0.1	44.7	99.2	300 000	[122]
1% Cu/*β*‐Mo_2_C	350 °C	0.1	11	40	300 000	[123]
Mo_2_C/*γ*‐Al_2_O_3_	300 °C	2.1	5.9	87.8	N/A	[124]
%5 Ru/CeO_2_	350 °C	0.1	16	31	120 000	[125]
RuNi/CeZr	350 °C	0.1	53	93	24 000	[126]
FeNi/CeZr	350 °C	0.1	13	60	24 000	[126]
1 wt.% K‐Mo_2_C/*γ*‐Al_2_O_3_	300 °C	2.1	24.3	73.5	3060	[127]
%5 Ru/Sm‐CeO_2_	350 °C	0.1	16	69	120 000	[125]
3.2% PtCo/CeO_2_	300 °C	0.1	9.1	92.3	N/A	[128]
Ni_2_P/SiO_2_	650 °C	0.1	58	84	12 000	[129]
Mo‐P‐SiO_2_	750 °C	0.1	69.1	85.1	12 000	[130]
3.2% PtCo/TiO_2_	300 °C	0.1	8.2	98.8	N/A	[128]
2D(*δ*)‐MnO_2_	850 °C	0.1	50	100	40 000	[131]

Transition metal‐based non‐precious catalysts are desirable due to their abundance as raw materials, and low cost. Copper‐based catalysts were found to be selective in the production of CO by RWGS reaction.^[^ ^132^, ^133^, ^134^, ^135^, ^136^, ^137^, ^138^, ^139^ ^]^ Mo‐based catalysts are also common for RWGS. In particular, Mo_2_C is widely studied^[^ ^51^, ^122^, ^140^, ^141^ ^]^ in many studies because they are active and selective in RWGS reactions.^[^ ^124^ ^]^ Potassium‐promoted molybdenum carbide catalyst supported on *γ*‐Al_2_O_3_ (K‐Mo_2_C/*γ*‐Al_2_O_3_) was recently shown to achieve 40.5% CO_2_ conversion and 98.2% CO selectivity at 2.1 MPa and 450 °C. The results of this study show that it is a low‐cost, stable, and highly selective catalyst for RWGS reactions.^[^ ^127^ ^]^ It has also been shown that molybdenum phosphide is a suitable catalyst for the RWGS reaction, and preserves its chemical structure in hydrogen up to 950 °C, important for its stability.^[^ ^130^ ^]^ In addition, phosphite‐containing catalysts showed high catalytic performance in studies such as dry reforming of methane (DRM), hydrodeoxygenation of guaiacol, and conversion of CO_2_ by RWGS reaction.^[^ ^142^, ^143^, ^144^ ^]^ In a different study, Mo_2_C also differentiated itself from other catalysts for CO_2_ conversion, with its dual functionality for H_2_ dissociation and C=O bond scission, and properties similar to reducible oxides.^[^ ^145^ ^]^

### 2.3 Catalysts for FTS

The syngas is catalytically converted to higher hydrocarbons by FTS, which is then converted into clean fuels, oils, or chemicals. Ru,^[^ ^146^ ^]^ Ni,^[^ ^147^ ^]^ Fe,^[^ ^148^ ^]^ and Co^[^ ^149^ ^]^ catalysts are the most preferred active metals in FTS. However, active metals Fe and Co are used as commercial FTS catalysts.^[^ ^150^ ^]^ Co‐based catalysts can be said to be the most suitable catalyst in gas‐to‐liquid conversion technologies due to their high efficiency and selectivity, low prices, as well as low WGS activities for syngas with a high H_2_/CO ratio.^[^ ^151^ ^]^ However, Fe‐based catalysts are preferred for low H_2_/CO ratio syngas because these catalysts have high intrinsic selectivity for the WGS reaction.^[^ ^152^ ^]^ The reaction temperature has great importance in the selection of the catalyst. FTS catalysts are operated using cobalt‐based or iron‐based catalysts at low temperatures (220–260 °C) and using iron‐based catalysts at medium temperatures (260–300 °C) or high temperatures (320–350 °C).^[^ ^153^ ^]^

**Table** **3** contains some recent studies on iron, cobalt, lanthanum, copper, and manganese catalysts used for FTS, including catalyst type, reactant conversion, and product selectivity. Also, this table summarizes the reaction conditions, CO conversion, and CH_4_, C_2_–C_4_, and C_5+_ fraction selectivity, as these reactants are important products from FT synthesis.

**Table 3:** Catalysts for converting syngas into hydrocarbons via FTS

Catalyst	Temperature	Pressure	Conversion			Selectivity			Reference
			CO	H_2_	CO_2_	CH_4_	C_2_–C_4_	C_5+_	
Co/*γ*‐Al_2_O_3_	230 °C	20 Bar	52.4	58.3	13.1	15.2	8.7	76.1	[154]
CeCo/*γ*‐Al_2_O_3_	230 °C	20 Bar	54.0	64.3	20.3	10.5	5.3	84.3	[154]
LaCo/*γ*‐Al_2_O_3_	230 °C	20 Bar	61.5	66.7	21.9	9.7	5.5	84.8	[154]
CeLACo/*γ*‐Al_2_O_3_	230 °C	20 Bar	62.9	67.9	22.1	9.6	5.4	85.0	[154]
CoRu/*γ*‐Al_2_O_3_	200 °C	20 Bar	20	‐	‐	10.6	‐	74.3	[155]
Fe	350 °C	2MPa	90.04	‐	‐	24.46	8.04	20.56	[156]
FeAl	350 °C	2MPa	97.16	‐	‐	25.96	10.30	19.94	[156]
Fe—Cu—Mn/AC	300 °C	2MPa	98	‐	‐	24	30		[157]
Co/PGNS	220 °C	1.8 MPa	70.6	‐	‐	12.3		86.8	[158]

### 2.4 Two‐Step CO_2_ Hydrogenation with Methanol Synthesis (MS) + Methanol to Olefins (MTO)

In the methanol‐based (CH_3_OH) two‐step conversion of CO_2_, the hydrogenation of CO_2_ to methanol is followed by a methanol‐to‐olefins (MTO) transformation. In the tandem catalytic process, first, CO_2_ and H_2_ are converted to CH_3_OH (Equation (2)) on a partially reduced oxide surface (e.g., Cu, In, and Zn) or over noble metals.^[^ ^159^ ^]^

When the studies carried out in recent years are examined, it is reported that direct conversion of CO_2_ to hydrocarbons can be achieved in one reactor with a CH_3_OH‐mediated method (**Table** **4**). In_2_O_3_/HZSM‐5 composite catalyst was shown in a study to perform much better for CO_2_ hydrogenation with higher activity and higher selectivity towards hydrocarbons while comparing other metal oxides combined with HZSM‐5 such as Ga_2_O_3_, Fe_2_O_3_, ZnO—Cr_2_O_3_, and ZnO—ZrO_2_.^[^ ^160^ ^]^ According to these studies, H‐ZSM‐5 is active for hydrocarbon oligomerization, isomerization, and aromatization, and adjusting the acidity of H‐ZSM‐5, promotes the production of gasoline and aromatics. In the studies carried out, the selective production of gasoline has been made from zeolites such as H‐ZSM‐5, H‐Y, H‐beta, and H‐MCM‐22, while aromatics have been predominantly produced from H‐ZSM‐5 zeolites. As explained in the studies, the stronger Brønsted acidity of H‐ZSM‐5 supports the aromatization reaction, while the weaker acidity of H‐beta or H‐MCM‐22 supports the isomerization reaction.^[^ ^161^, ^162^ ^]^ The content of the final product can be adjusted by adjusting the acid strength and pore size of the zeolites. Oxygen cavities on the surface of In_2_O_3_ are proposed to activate CO_2_ and hydrogen, resulting in the formation of methanol. Afterward, C—C coupling takes place in the zeolite pores, producing gasoline‐grade high‐octane hydrocarbons. The combination of these two components plays an important role in slowing down the unwanted RWGS reaction and providing a high selectivity for high‐carbon fuels.^[^ ^160^ ^]^

**Table 4:** Performance of some tandem catalysts for methanol‐mediated CO_2_ conversion to hydrocarbons

Catalysts	Temperature [°C]	Pressure [MPa]	CO_2_ Conversion [%]	Selectivity [%]	Targeted Product	Reference
CuZnZr@(Zn‐)SAPO‐34	400	2	20	72	C_2_–C_4_	[166]
ZnO/ZrO_2_‐ZSM‐5	340	3	9	40	C_2_–C_4_	[167]
ZnZrO/ZSM‐5	320	4	14	73	Aromatics	[168]
ZnO—ZrO2/H‐ZSM‐5	340	4	16	76	Aromatics	[169]
ZnCrOx‐ZnZSM‐5	320	5	19.9	81.1	Aromatics	[170]
Cr_2_O_3_/H‐ZSM‐5	350	3	34.5	75.9	Aromatics	[171]
Cr_2_O_3_/Zn‐ZSM‐5@SiO_2_	350	3	22.1	70.1	Aromatics	[172]

Lower reaction temperatures and higher reaction pressures positively affect the synthesis of CH_3_OH. At the same time, these stated conditions have in some cases increased the formation of by‐products such as formic acid, methane, and formaldehyde, and therefore a selective catalyst is required that is also stable under such conditions.^[^ ^163^ ^]^

Hydrogenation of CO_2_ based on the methanol (CH_3_OH) reaction can be performed by combining two sequential reactions on a tandem catalyst.^[^ ^36^ ^]^ As mentioned earlier, CO_2_ and H_2_ are first converted to CH_3_OH via a CO or formate pathway, either on the partially reduced oxide surface (e.g., Cu/ZnO/Al_2_O_3_, In_2_O_3_/ZrO_2_) or noble metals.^[^ ^11^ ^]^ It is then dehydrated or combined with zeolites or alumina. Therefore, tandem catalysts are formed that can convert CO_2_ into high‐value C_2_+ compounds, such as DME, light olefins, and gasoline. An effective catalyst to be used for these reactions must be active for both CH_3_OH synthesis and dehydration/C—C coupling. In the C_2+_ hydrogenation reactions of CO_2_, the reactions of CO_2_ to CH_3_OH and CH_3_OH to C_2_+ compounds take place on bifunctional catalysts at 200–300 and 400 °C, respectively. Accordingly, it is necessary to investigate the reaction conditions, catalyst properties, and catalytic performance of the products CO_2_ to CH_3_OH and CH_3_OH to C_2_+.

Considering the catalysts used for the synthesis of CH_3_OH from the synthesis gas used on an industrial scale, Cu/ZnO/Al_2_O_3_ catalysts were widely studied.^[^ ^163^ ^]^ For the methanol‐to‐olefins step, medium porous zeolite/microporous materials are preferred to produce C_5_–C_11_ hydrocarbons, while small porous molecular sieves are preferred to produce C_2_–C_4_ hydrocarbons. ZSM‐5 is commonly used for converting methanol to gasoline (MTG) and SAPO‐34 molecular sieves for methanol to olefins (MTO).^[^ ^164^, ^165^ ^]^ SAPO‐34 molecular sieves are particularly preferred to achieve high selectivity in the production of light olefins.^[^ ^11^ ^]^

### 2.5 Catalysts for Methanol Synthesis from CO_2_

Recently, there have been significant developments involving copper (Cu)‐ and indium (In)‐based catalysts for methanol synthesis.^[^ ^173^, ^174^ ^]^ Cu catalysts (Cu/ZnO/A1_2_O_3_) are industrially used for the synthesis of methanol from CO_2_/H_2_ or syngas (CO/CO_2_/H_2_) under the operating conditions of 220–300 °C and 50–100 bar temperature and pressure, respectively.^[^ ^175^, ^176^ ^]^ Also, Pt and Pd are effective, especially in the low‐temperature and pressure synthesis of methanol from CO_2_.^[^ ^132^, ^133^ ^]^

The Cu catalyst is one of the preferred catalysts of choice to produce methanol from CO_2_. Because methanation is partially prevented by the reaction, the standard Cu/ZnO/Al_2_O_3_ catalyst is preferred in industrial methanol production. In a study using CuO/ZnO catalysts, filament‐like ZnO and rod‐like ZnO synthesized by the hydrothermal method were used as components. These newly developed catalysts were tested in the hydrogenation of CO_2_ to methanol and compared with the CuO/ZnO catalyst prepared by the conventional method. The activities of these catalysts were found to be strongly dependent on the ZnO morphology. The catalyst prepared with filament‐like ZnO exhibited the best activity with 78.2% methanol selectivity at H_2_/CO_2_ = 3, 240 °C, 3.0 Mpa.^[^ ^177^ ^]^

Cu—ZnO—Ga_2_O_3_/SiO_2_ and LaCr_0.5_Cu_0.5_O_3_ have also been shown to demonstrate higher catalytic performance towards methanol with selectivity of 99.1% and 90.8% and conversion of 2.0% and10.4% at 250 and 270 °C, respectively.^[^ ^178^, ^179^ ^]^ However, these mixed oxides did not maintain a stable structure for long reaction cycles^[^ ^180^ ^]^ that In_2_O_3_, which is used as a catalyst in the conversion of CO_2_ to methanol, has good catalytic activity and can be further promoted with Pd, Ni, etc.^[^ ^181^, ^182^, ^183^, ^184^, ^185^, ^186^ ^]^

There are several recent papers about methanol production that Cu—ZnO composites with less than 30% CO_2_ conversion and CH_3_OH selectivity ranging between 30% and 70%. The reaction conditions of temperature, pressure, and H_2_/CO_2_ ratio were 220–300 °C, <5 MPa and 3, respectively have been considered.^[^ ^187^, ^188^ ^]^ If the pressure and the H_2_/CO_2_ molar ratio are increased to 36 MPa and 10, respectively, on a Cu—ZnO—Al_2_O_3_ catalyst; 95.3% CO_2_ conversion and 98.2% methanol selectivity are obtained.^[^ ^189^ ^]^

In a study to produce methanol from CO_2_, Fluorinated Cu/Zn/Al/Zr hydrotalcite was synthesized using (AlF_6_)^3−^. In this study, it was stated that the amount and strength of adsorption applied to CO_2_ has an effect on methanol production and is a guide for the development of efficient catalysts.^[^ ^190^ ^]^ Rungtaweevoranit et al. developed a catalyst where Cu nanocrystal (NC) is encapsulated in a Zr(IV)‐based MOF, designated as Cu⊂UiO‐66; UiO‐66 [Zr6O4(OH)4(BDC)6, BDC = 1,4‐benzenedicarboxylate] for hydrogenation of CO_2_ to methanol. They found that Cu⊂ UiO‐66 created an eightfold increased catalytic activity compared to the Cu/ZnO/Al_2_O_3_ catalyst while maintaining 100% selectivity towards methanol.^[^ ^191^ ^]^

Indium‐based catalysis has been accepted among the alternatives for the conversion of CO_2_ to methanol. It was found that a pure In_2_O_3_ catalyst at 330 °C and 5 MPa could convert approximately 7% of CO_2_ to CH_3_OH with selectivity above 39%.^[^ ^181^ ^]^ In another study with In‐based catalysts, a Pd/In_2_O_3_ catalyst at 300 °C and 5 Mpa reaction conditions with many interfacial regions and oxygen vacancies (to increase CO_2_ adsorption) achieved more than 20% CO_2_ conversions and over 70% methanol.^[^ ^174^ ^]^ In another study, O. Martin et al. prepared a stable In_2_O_3_/ZrO_2_ composite catalyst for methanol synthesis, and this catalyst showed 1000 recycling capabilities.^[^ ^192^ ^]^ In_2_O_3_ nanoparticles overcome the RWGS reaction and offer 100% selectivity to methanol at all temperatures, while Cu—ZnO—Al_2_O_3_ provides 47% methanol yield due to the formation of the RWGS reaction.^[^ ^193^ ^]^

Ni—Ga and MoP are also among the recently developed catalysts targeting hydrogenation of CO_2_ to methanol. For example, at 10 bar and 200−270 °C using Ni—Ga, Au—Ni—Ga, Co‐Ni—Ga, and Cu—Ni—Ga catalysts, methanol can be synthesized with CO_2_ conversion of 0.35–1,2, 0.2–0.8, 0.3–1, and 0.4–1.6 and selectivity of 55–45, 70–50, 65–55, and 65–55, respectively. Also, Au is seen to improve turnover frequency through a promotion effect.^[^ ^194^ ^]^ Ni_5_Ga_3_ catalysts were synthesized for the hydrogenation of CO_2_ to methanol and the effects of surface oxidation/reductions on Ga catalytic performance by ex situ and in situ characterization techniques were investigated. At the end of the study, it was found that the presence of amorphous Ga_2_O_3_ on the surface of metallic nanoparticles was involved in methanol synthesis and promoted CO_2_ activation rather than preventing CO_2_ reduction.^[^ ^195^ ^]^ Molybdenum phosphide (MoP) catalysts have been recently developed, showing a stable performance for methanol synthesis catalysts that are not affected by the ratio of CO and CO_2_ in the feed. The formate binding strength over MoP catalysts was found to be a determining factor in controlling feed‐agnostic activity for methanol synthesis.^[^ ^196^ ^]^ When zirconia (ZrO_2_) was used as a support for MoP nanoparticles, methanol selectivity, and conversion rate were found to be improved greatly over other metal oxide supports.^[^ ^197^ ^]^

A Ga‐Pd/SiO_2_ catalyst with higher performance compared to standard Cu/ZnO/Al_2_O_3_ was developed, they stated in their study that the CH_3_OH selectivity of the GaPd_2_/SiO_2_ catalyst is two times higher than that of the conventional copper catalyst.^[^ ^135^ ^]^ One study implied that Pd‐Cu bimetallic catalysts exert a strong synergistic effect on the selective incentive for the formation of CH_3_OH in CO_2_ hydrogenation in the ratio Pd/(Pd+Cu): 0.25–0.34 and reaction conditions of 523K, 4.1 MPa.^[^ ^136^ ^]^ Noble metal‐based catalysts such as Ga—Pd, Au—CeO_X_/TiO_2,_ and Pt—MoO_X_/Co‐TiO_2_ have been developed and used to produce CH_3_OH from CO_2_ at low pressure or low temperature.^[^ ^135^, ^137^, ^138^ ^]^ Similarly, the synthesis of methanol was obtained at 255 °C and 0.1 MPa with the catalyst series containing In_2_O_3_ such as Ni—In—Al/SiO_2_ and La—Ni—In—Al/SiO_2_.^[^ ^198^, ^199^ ^]^

## 3 Nanoreactors

Nanoreactors are an important and innovative means to mimic processes occurring in nature.^[^ ^76^, ^200^ ^]^ Nanoreactors first appeared in the 1990s and have become increasingly more significant in catalytic applications.^[^ ^201^ ^]^ Nanoreactors refer to compartmentalized nanostructures to perform catalytic functions under controlled local environments, analogous to cellular organelles in a living organism, with the aim of increasing reaction rates and production efficiency. Nanoreactors have some advantages over conventional catalysts. These include performing parallel chemical reactions, producing less undesirable products, and increasing the catalytic performance due to large surface‐to‐volume ratios.^[^ ^200^ ^]^

The size, shape, particle composition, and microenvironment of the structure contained within a nanoreactor play a significant role in chemical reactions and can provide renewed stability and selectivity.^[^ ^202^ ^]^ In the most general sense, nanoreactors are divided into two groups: they are natural and synthetic nanoreactors. Natural nano‐reactors contain protein‐based bacterial microcompartments, protein cages, and viruses. Synthetic nanoreactors, on the other hand, have more types and include molecules, macromolecules, nanostructures, and porous solids. A molecular nanoreactor is formed by the accumulation of several molecules together and forming a cavity for a chemical reaction. For macromolecular nanoreactors, polymers are preferred in the form of large single molecules that are hollow or in self‐assembled structures with one or more cavities. In core‐shell nanostructures, variable‐core structures embedded in a hollow shell have been greatly enhanced. If the outer shell of the nanostructure is permeable to the reactants, these structures become nanoreactors with variable catalytic cores. The commonly preferred compounds in the porous solids group are the porous structures of silicate and zeolite. Zeolites are composed of aluminosilicate and are porous compounds. The sizes of cavities in silicates and zeolites range from a few angstroms to a few nanometers.^[^ ^81^, ^203^ ^]^

### 3.1 Encapsulated Materials

The creation of a core‐shell nanostructure can protect the active site of a catalyst from deactivation through agglomeration, as is often observed for metallic nanoparticle catalysts or surface‐coating hybrids.^[^ ^204^, ^205^ ^]^ A majority of tandem catalysis schemes use supported metal nanoparticles (SMN), sometimes in combination with zeolites. However, these innovative encapsulated materials are more useful than SMN in the protection of the metallic core, where the catalytic reaction takes place, by the porous shell. This shell protects the metallic core from agglomeration or coking that occurs in traditional SMN formulations.

**Figure 3:**
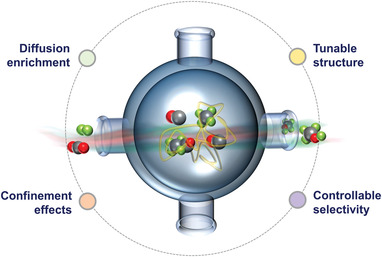
A schematic representation of some unique features of YolkShell.^[^ ^213^ ^]^

Nanoreactors can also be designed as yolk@shell materials.^[^ ^206^ ^]^ Compared to the core‐shell architecture, the yolk‐shell has a void between the “yolk” and the metallic core, associated with improved catalytic performance.^[^ ^200^, ^205^, ^207^, ^208^, ^209^, ^210^, ^211^ ^]^ Yue et al. have devised the synthesis of a new type of yolk‐shell magnetic mesoporous silica microspheres. The resulting yolk‐shell Fe_3_O_4_@SiO_2_@hollow mSiO_2_ microspheres have open and regular mesoporous channels (2.2 nm) and a controllable void space (320–430 nm). These large void space catalysts exhibited excellent catalytic performance with high conversion and selectivity.^[^ ^212^ ^]^ Yolk shell structures provide several advantages and three of them stand out.^[^ ^213^, ^214^ ^]^ 1) the total exposure time of the active center can be adjusted to the desired values for some degree of catalytic efficiency and stability; 2) the volume of the void can expand for catalytic reaction and mass transfer to occur; 3) Modifications to the shell, yolk, void or a combination of these provide a flexible and dynamic nature of catalytic efficiency, stability, and recyclability. The design and development of YS achieve these goals, and many special features of YS are emerging, as briefly described in **Figure** **3**. Many different synthesis strategies have been developed to prevent deactivation, including Atomic Layer Deposition (ALD), Strong Metal Support Interaction (SMSI), non‐hydrolytic sol‐gel, and core‐shell methods.^[^ ^215^ ^]^ Also, encapsulating the active phase with a protective and porous shell material prevents normal deactivation states. The benefits of the structures of catalysts using this morphology are better understood by the studies. Its most important benefit is confinement effects. These confinement effects prevent carbon deposits from accumulating and sintering while providing a homogeneous reaction environment, both as a protective and performance‐enhancing feature.^[^ ^216^, ^217^ ^]^ At the same time, it is easier for more complex reactions to occur due to increased internal pressure. These nanoreactors exhibit improved performance due to simultaneous reaction capability, higher product selectivity, and larger active surface area compared to conventional materials.^[^ ^206^, ^218^ ^]^

If we look at their general properties, these particles consist of a metal or metal oxide core within an inorganic or polymeric shell; Silica or carbon are commonly preferred choices, as they are extremely stable, abundant, and inert.^[^ ^219^ ^]^ The performance of this morphology depends on several factors. These factors are particle size, shell thickness, and sample homogeneity, which can be adjusted by synthetic methods.

With the increasing interest in YS catalysts, more advanced and complex YS structures have been developed.^[^ ^220^, ^221^, ^222^, ^223^ ^]^ These complex structures range from “basic” single‐encapsulated cores to structures with multiple encapsulated cores called “raspberry cores” and are even present in structures with a single multi‐shelled core.^[^ ^221^, ^224^ ^]^ In general, these more advanced variations derived from the single encapsulated core exhibit more advanced performance due to their structural diversity. For example, multiple cores offer higher active surface area.^[^ ^225^ ^]^ Although each generated variant has its advantages, no variant can be described as a superstructure because of the possible different needs for each chemical process. Therefore, YS particles tailored to the reaction are required.^[^ ^211^, ^226^, ^227^ ^]^

### 3.2 Applications of Nanoreactors for CO_2_ Hydrogenation

Based on the studies reported in the literature, the yolk@shellYS structure can be said to be very effective in producing alcohols from CO_2_ with a Cu‐encapsulated catalyst.^[^ ^191^, ^228^, ^229^ ^]^ In a study, ultra‐small Cu/ZnO_x_ nanoparticles were produced in MOF cavities to perform CO_2_ hydrogenation. These Cu/ZnO_x_@MOF catalysts obtained showed very high activity with 100% selectivity to methanol and high stability over 100 h.^[^ ^230^ ^]^ The catalyst used in another study is synthesized by cobalt nanoparticles onto amorphous silica (Co@Si_x_) to form Co—O—SiO_n_ that stabilizes methoxy (*CH_3_O) species in CO_2_ hydrogenation. By optimizing the cobalt‐to‐silica ratio, they achieve better performance than the noble metal catalysts as well as the supported copper catalysts used to convert CO_2_ to methanol.^[^ ^231^ ^]^ They have obtained methanol selectivity of 70.5%, CO_2_ conversion of 8.6% at 320 °C, 2 MPa, and methanol productivity of 3.0 mmol g_cat_^−1^h^−1^ for Co@Si0.95_._ In a different study, core‐shell catalysts with Cu and Cu/ZnO nanoparticles coated with mesoporous silica shells (Cu@m‐SiO_2_ and Cu/ZnO@m‐SiO_2_ nanocatalysts) were produced and used for the hydrogenation of CO_2_ into methanol. After 168 h of reaction, the CO_2_ conversion and CH_3_OH selectivity decreased by 1.6% and 1.0%, respectively, compared to the initial values. They found that the core‐shell Cu/ZnO@m‐SiO_2_ catalyst provided maximum CH_3_OH yield (153.9 g kg cat^−1^ h^−1^) with high stability.^[^ ^228^ ^]^

In another study, a new core‐shell structured CuIn@SiO_2_ catalyst was produced by the solvothermal method and used to catalyze the hydrogenation of CO_2_ into methanol. This new catalyst has improved CO_2_ adsorbing ability to provide more CO_2_ for hydrogenation reaction and 9.8% CO_2_ conversion and 78.1% CH_3_OH selectivity are obtained. According to the authors, this newly prepared CuIn@SiO_2_ catalyst offers high catalytic stability and catalytic performance due to core‐shell formation.^[^ ^232^ ^]^ The catalytic performance of the catalyst was 9.8% CO_2_ conversion, 78.1% CH_3_OH selectivity, and 13.7 mmol_CH3OH_ h^−1^ gcat^−1^ space‐time yield at 280 °C and 3 MPa.

**Figure 4:**
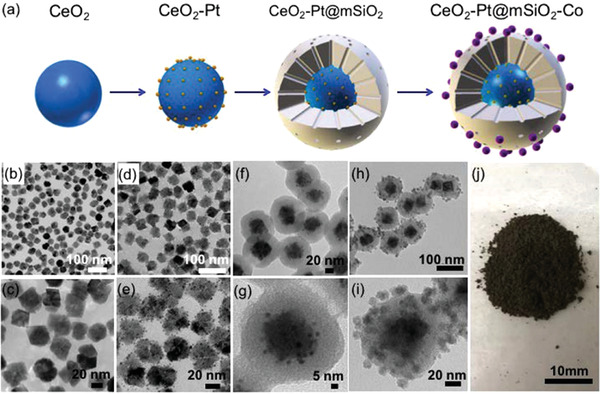
Synthesis and TEM images of the CeO2—Pt@mSiO2—Co tandem catalyst. a) Illustration of the synthetic process. TEM images of each step: b,c) CeO2 nanoparticles, d,e) Loading of Pt nanoparticles on CeO2, f,g) silica shell coating on CeO2—Pt, h,i) settling of Co nanoparticles on CeO2—Pt@mSiO2, and j) CeO2—Pt@mSiO2—Co nanoparticles. Reproduced with permission.^[^ ^233^ ^]^ Copyright 2017, ACS Publications.

Generally, catalysts are prepared by conventional methods of co‐precipitation, impregnation, or physical mixing and may contain multiple components, including various supports. They can show significant uncertainties in the spatial arrangement of active areas. In a recent study, a well‐defined CeO_2_—Pt@mSiO_2_—Co core‐shell catalyst was prepared (**Figure** **4**). Here, the synthesis of C_2_–C_4_ hydrocarbons from CO_2_ conversion with a two‐step tandem reaction is targeted. While the RWGS reaction produces CO on a CeO_2_/Pt interface, the FTS process takes place at the Co/mSiO_2_ interface to form C—C coupled products. According to the study, catalyst synthesis has four steps. The first step is the synthesis of well‐dispersed and uniform CeO_2_ nanoparticles, as seen from the TEM images (Figure 4b,c). The second step is to load Pt nanoparticles onto pre‐prepared CeO_2_ by the Pt overgrowth method (Figure 4d,e). In the third step, a sol‐gel method is preferred to coat a mesoporous SiO_2_ shell on the CeO_2_‐Pt core (Figure 4f,g). The cobalt‐hexane solution was added to CeO_2_‐Pt@mSiO_2_ powder by mixing slowly, thus a uniform cobalt nanoparticle distribution on the silica shell was obtained (Figure 4h,i). With this four‐step synthesis, CeO_2_—Pt@mSiO_2_—Co tandem catalysts were obtained (Figure 4j). They tested the catalyst under 0.6 MPa pressure and 250 °C reaction conditions. They found out that the CO_2_ conversion (%), CO selectivity (%), CH_4_ selectivity (%), and C_2_–C_4_ (%) selectivity values as 2.0, 78.0, 60.0, and 40.0, respectively. In this catalyst, CO_2_ and H_2_ were converted to CO via the Pt/CeO_2_ interface, and the other interface, Co/mSiO_2_, produced C_2_–C_4_ hydrocarbons by a subsequent Fischer–Tropsch process.^[^ ^233^ ^]^

Hollow spherical dual‐layer Co@hsZSM5@Pt nanocatalysts were found to be highly effective in converting CO_2_ with 46% hydrocarbon selectivity of C_2+_ by the Fischer–Tropsch reaction at 400 °C and 20 bar. According to the tandem hydrogenation mechanism, Pt on the outer surface also carries out the initial conversion of CO_2_ to CO (RWGS), and the inner Co is known to convert CO to C_2+_ and methane (FTS).^[^ ^234^ ^]^

Another hollow ZSM‐5 zeolite nanoparticle with Fe_3_O_4_@MnO_2_ was used to convert syngas directly into aromatic‐rich gasoline. Approximately 70.0% CO conversion and selectivity of 73.5% for gasoline‐grade hydrocarbons at 320 °C and 4.0 MPa were obtained. The article mentioned that forming a hollow structure shortens the diffusion length and thus increases the stability of zeolite nanoparticles.^[^ ^235^ ^]^

In a study, 24.1% CO_2_ conversion and 86.3% selectivity of light olefins were obtained with the ZrO_2_—ZnO—(CeO_2_)_2_/SAPO‐34 catalyst at T  =  350 °C, p  =  2.6 MPa and GHSV  =  6000 mL (gca h)^−1^. Also, the catalyst with a strong CO_2_ and H_2_ adsorption capacity was obtained by incorporation of CeO_2_ into Zr—Zn oxide which was greatly increased concentration of oxygen vacancies on the catalyst surface.^[^ ^236^ ^]^

In a different study, methanol and dimethyl ether (DME) were produced at between 180–260 °C by 10Fe‐10Cu/silica‐aluminosilicate core‐shell and 10Fe‐10Cu/silica‐aluminosilicate infiltrated catalysts from CO_2_ with the assistance of a magnetic field.^[^ ^237^ ^]^ While the percentage of CO_2_ conversion was found to be similar for both catalysts, the core@shell catalyst was more selective for methanol while the infiltrated catalyst was more selective for DME. The researchers tested both catalysts at a temperature range of 180–260 °C and a total pressure of 10 bar. Both catalysts produced CO above 220 °C and even at 260 °C, the CO_2_ conversions in both catalysts remained below 10%. Jiang et al. developed a fixed‐bed process using a series of Pd/In_2_O_3_/SBA‐15 catalysts to convert CO_2_ to methanol. The 10% Pd/In_2_O_3_/SBA‐15 catalyst exhibited excellent catalytic performance at 260 °C, 5 Mpa and 15 000 cm^3^ h^−1^ g_cat_^−1^, with a methanol selectivity of 83.9%, a CO_2_ conversion of 12.6%, and a catalyst yield of 1.1×10^−2^ mol h^−1^ g_cat_^−1^. According to the results obtained, the oxygen vacancies in In_2_O_3_ increased with the addition of Pd, and they reported that this process facilitated the activation of CO_2_. With these, the H needed to hydrogenate CO_2_ into methanol was provided, with which Pd could easily separate large amounts of H_2_.^[^ ^238^ ^]^ In light of the data obtained from this study, the catalysts used can be evaluated in nanoreactor studies.

As described above, many valuable chemicals and fuels can be produced from CO_2_ with tandem catalysts especially when nanoreactor configurations are employed. While producing some chemicals and fuels from CO_2_ provides economic benefits, it also provides benefits for the environment by reducing the CO_2_ level in the atmosphere. In recent studies, especially tandem and nanocatalyst technologies are combined, and the effective use of yolk‐shell and core‐shell structures in CO_2_ conversion is described. Although many advances have been made academically and industrially, CO_2_ conversion rates are not industrially sufficient. In order to overcome this deficiency, there is a need to develop tandem nano‐catalysts with a higher conversion rate of core‐shell or yolk‐shell structure. To achieve this, the relationship between the design and performance of nanocatalysts must be understood. Studies in which characterization techniques are applied to see the structural changes that occur during catalytic reactions should be emphasized.

## 4 Conclusions and Outlook

Nanoreactors offer a way to achieve precision in catalyst synthesis and some examples of the nanoreactor approach have been demonstrated for tandem catalytic conversion of CO_2_ to C—C coupled products by synthesis of a core‐shell or yolk‐shell structure. Nanoreactors can be used to perform multiple reactions operating in tandem to achieve high selectivity and rates for desired products, such as in combined RWGS + FTS or MS + MTO pathways. Here, since the porosity of the outer shell can be customized to adjust the diffusion rate of the reactants, it provides the advantage of better controlling the reaction rate and catalytic performance. However, more studies are needed on these materials to reveal their true potential. In addition to studies on lifetime and deactivation, improvements can be made for effective and scalable catalyst production. In particular, the necessity of precision manufacturing makes the commercial production of nanoreactors a challenge that must be addressed for commercial implementation. First, the relationship between structure and performance needs to be understood for the design of nanocatalysts. To see the structural changes of nanocatalysts that occur during catalytic reactions, characterization techniques should be applied. Second, there are two very important steps to increase the efficiency of catalytic CO_2_ conversion, these are to reduce the activation barrier of CO_2_ and accelerate the formation of intermediates. The calculations of the inclusion of defects and vacancies in the structure of nanocatalysts are critical for the efficiency of catalytic CO_2_ conversion and this issue is confirmed by some theoretical calculations. An important method for accelerating CO_2_ conversion is to incorporate single‐atom centers into nanocatalysts that have high catalytic activities for CO_2_ activation and conversion.

## Conflict of Interest

The authors declare no conflict of interest.
